# Context-dependent memory in the real world: the role of frequency and context dwell time

**DOI:** 10.3389/fpsyg.2024.1489039

**Published:** 2025-01-28

**Authors:** Yura Choi, Kawon Kim, Sunwoo Moon, Geunseong Jung, Jae-Hyuk Cha, Hyungwook Yim

**Affiliations:** ^1^Department of Cognitive Sciences, Hanyang University, Seoul, Republic of Korea; ^2^Department of Psychology, University of South Dakota, Vermillion, SD, United States; ^3^Department of Computer Science, Hanyang University, Seoul, Republic of Korea

**Keywords:** context-dependent memory, experience sampling, episodic memory, context, encoding specificity principle

## Abstract

We investigated the context-dependent memory effect outside of the laboratory in order to examine whether the effect impacts everyday memory retrieval. We also examined various factors that may interact with the context-dependent memory effect such as frequency and context dwell time. In the experiment, we used a smartphone app to track participant’s GPS locations for 5 weeks. Participants, during their daily lives, were then asked to recall their locations at a specific date and time by choosing from all locations visited in the previous 5 weeks. Results demonstrated the existence of the context-dependent memory effect in a real-world setting, with low-frequency locations showing a stronger context-dependent memory effect—benefiting more from the matched context. We also found that for low-frequency locations, the benefit of the context-dependent memory effect increased as the participant spent more time in the context (context dwelling time). The study provides a novel way to examine the context-dependent memory effect outside of the laboratory, which not only enables researchers to measure an individual’s genuine memories in a more ecologically valid way, but also investigates factors that would be challenging to examine in the laboratory.

## 1 Introduction

The encoding specificity principle (Tulving and Thomson, 1973) has led memory researchers to focus not only on the properties of the items at encoding but also on the context at encoding and retrieval. Tulving and Thomson (1973) emphasized the importance of the context in memory arguing that memory performance may improve when individuals are in a context similar to where the event was initially encoded. Their principle implied that the ability to remember focal information accurately is significantly influenced by the relationship between the encoding and retrieval contexts. For instance, when we listen to a particular song, it can evoke memories of specific moments we experienced with that song being in the background (or context). This is because the music, as a context of the memory, reinstates familiar experiences, allowing us to recall the moment as if we were reliving it. This phenomenon is referred to as the *context-dependent memory* effect, where memory performance enhances when the context during encoding matches the context during retrieval.

A seminal experiment by Godden and Baddeley (1975) has been a well-known controlled study for showing the context-dependent memory effect. In this study, participants either learned words underwater or on land. Subsequently, they were divided into groups to be tested in the same context as learning or in a different context. The results demonstrated enhanced memory performance when the contexts matched (i.e., studied on land and tested on land, or studied underwater and tested underwater), providing significant evidence that context is automatically stored during encoding and acts as a cue during retrieval, which improves memory accuracy.

The importance of context in episodic memory has led to exploring various forms of contexts in controlled laboratory settings. In general, the concept of “context” encompasses all relevant surrounding information excluding the focal information itself. Researchers have typically conducted experiments focusing on external contextual features (also see an extensive review by Smith and Vela, 2001). For example, Burri (1931) examined context by comparing performances in the presence and absence of audiences. Studies have examined the effects of context on memory performance from simple elements such as background colors or images (Dulsky, 1935; Isarida and Isarida, 2007; Murnane et al., 1999; Rutherford, 2004), to a more broader sense of contexts, such as distinctive rooms, places, or locations (Bjork and Richardson-Klavehn, 1989; Canas and Nelson, 1986; Coveney et al., 2013; Eich, 1985; Fernandez and Glenberg, 1985; Godden and Baddeley, 1975; Koens et al., 2003). Not only memory but also problem-solving has been investigated for the effects of context change (e.g., Beda and Smith, 2018). Moreover, various factors have also been studied as a single context, such as chewing gum, posture, background music, and odor (e.g., Balch et al., 1992; Cann and Ross, 1989; Isarida et al., 2014; Isarida et al., 2017; Johnson and Miles, 2008; Rand and Wapner, 1967; Schab, 1990; Smith, 1985). Additionally, internal or psychological features have been examined as context. For example, Smith (1995) proposed the concept of mental context, which includes not only the surrounding environment but also the participant’s mood, mental state, physiological events, and other incidental factors. Relatedly, memory studies have defined context by the mental state associated with the presence or absence of medication (Eich, 1985), by comparing two physiological states such as heart rate at rest or while exercising (Miles and Hardman, 1998), by mental context such as imagining a context (Chu et al., 2003; Masicampo and Sahakyan, 2014), and by mood context (Lewis and Critchley, 2003).

Despite the fact that numerous studies have examined the context-dependent memory effect, studies have predominantly been confined to controlled laboratory settings. Therefore, it is possible that the results from the laboratory-based studies may not truly capture the complex and dynamic nature of real-life situations, which results in low ecological validity. There can be at least three major limitations of the laboratory-based studies. First, the design of the memory tasks used in the laboratory is artificial compared to memory retrievals in everyday life. For example, unlike laboratory memory experiments, everyday memory encoding/retrieval seldom happens in multiple trials over a short period of time, and the contents are unlikely to be a set of random words or images. There have been attempts to resolve the shortcomings of laboratory-based experiments by using virtual reality (VR) technology (e.g., Chocholáčková et al., 2023; Shin et al., 2021). However, these studies do not fully address the limitations. Second, laboratory experiments usually present the target with the context at the same time. For example, the background of the screen (i.e., context) is presented with the word (i.e., item) together when the participant is tested for their memory. However, in our daily lives memory retrieval can happen at various points in a given context. For example, retrieving a shopping list can happen right after entering the grocery store or after 30 min of entering the store. Variability in the testing time not only reflects the dynamics of everyday memory retrieval more closely, but also provides a way to examine when the same context benefits memory performance in the context-dependent memory effect. Finally, laboratory experiments do not incorporate the dynamic structure of the environment in everyday life. The majority of the studies investigating context-dependent memory in the laboratory categorize the contexts in a binary manner (e.g., on land vs. underwater), and compare them as congruent or incongruent during the testing phase. Moreover, in contrast to the contexts and items encountered in everyday life, the contexts are treated equally without considering the properties of the context (e.g., how frequently one context or target item is experienced in the past).

In the current study, therefore, we examined context-dependent memory outside of the laboratory to address these concerns. To capture the participants’ daily lives, we utilized Experience Sampling Methods (ESM) via a smartphone app that tracked the participants’ location. The method provides the benefit of continuous and automated data collection, which avoids selective or biased sampling, and enhances ecological validity. ESM studies have provided interesting insights into how the human memory system works in real-life situations (Dennis et al., 2019; Laliberte et al., 2021; Yim et al., 2024). However, to our knowledge, there has been no specific research investigating the context-dependent memory effect outside of the laboratory.

## 2 Methods

To examine how context affects memory retrieval in everyday life, we collected participants’ location data passively using a smartphone app for five consecutive weeks. Subsequently, participants were cued with a certain date and time via their smartphones in their daily lives, and were asked to recall the location labels (i.e., target) they had visited during the data collection phase. There were two context conditions, where we considered context as the general features of the cued location, such as the atmosphere, ambient noise, and visual and spatial arrangement. In the congruent context condition, the context in which the participants were given the memory test (i.e., memory retrieving context) matched the context of the to-be-retrieved location label when it was encoded during the data collection phase (i.e., memory encoding context). In the incongruent context condition, the memory retrieving context and the memory encoding context did not match.

### 2.1 Participants

Fifty students (33 females, *M* = 21.34 years, *SD* = 2.18 years) from Hanyang University (Seoul, Republic of Korea) participated in the current study.^1^ The sample size was determined based on previous studies that examined episodic memory using experience sampling methods (e.g., Laliberte et al., 2021).^2^ Participants received a compensation of up to 120,000 KRW (approximately 100 USD) based on the number of test trials that they responded during the two-week online memory test phase (i.e., 12 questions per day, 168 questions in total). The research was approved by the Institutional Review Board at Hanyang University (HYU-2022-060).

### 2.2 Design and procedure

The experiment started with a five-week data collection phase followed by a two-week online memory test phase and a one-day post-survey. The post-survey phase was administered within a week after completing the online memory task, which involved tasks such as location identification, psychological similarity measurement, and frequency survey. The tasks were related to a separate research project to investigate the relationship between subjective psychological space and memory, which will be presented elsewhere (see Choi et al., 2023 for the details of the procedures).

#### 2.2.1 Phase 0: preparation for data collection

Participants visited the laboratory and installed smartphone apps for GPS tracking (i.e., Traccar),^3^ and for receiving webpage links to the online memory test (i.e., Telegram). The GPS tracking app was set to collect GPS locations every 60 s, and participants were told to keep the app active throughout the entire 7-week period. To ensure uninterrupted GPS data collection, participants were asked to maintain a consistent Wi-Fi/data connection, and regularly charge their phones to prevent battery drainage.

#### 2.2.2 Phase 1: data collection

The five-week data collection phase started on the first Monday and ended on the fifth Sunday during the seven-week experiment period. GPS locations were uploaded to the server immediately through Wi-Fi or data connection. The app log was monitored every morning by the experimenter to ensure that the data was collected every 60 s.

#### 2.2.3 Phase 2: online memory test

After the five-week data collection phase, the online memory test started on the sixth Monday and lasted for two full weeks. The test trials were based on the first 4 weeks of data, excluding data collected on the fifth week to incorporate a one-week retention interval before the online memory test. In their daily lives, participants received a webpage link to the online test page via the Telegram app 12 times a day between 10 am and 10 pm. The test page link was sent within every hour slot (e.g., 10:00 am–10:59 am) at a random minute, resulting in sending a total of 168 trials during the two-week test phase.

Test trials were generated by extracting stationary points from the raw GPS data, which was sampled every 60 s. Stationary points were defined when the participant stayed within a 50-meter radius for more than 15 min. Therefore, all events that were examined in the current study were events when the participant was not moving, and all moving events (e.g., riding a subway to school) were not considered due to the technical difficulties of defining the moving event (or moving path) without directly verifying the event through the participant’s memory. As stationary points were individual events that occurred at a certain location point, we also extracted location points. We defined location points by clustering the stationary points using the DBSCAN algorithm in the scikit-mobility Python package (Pappalardo et al., 2022) with setting the epsilon parameter to 35 meters. The median GPS coordinates were used for both stationary and location points.

**Figure 1:**
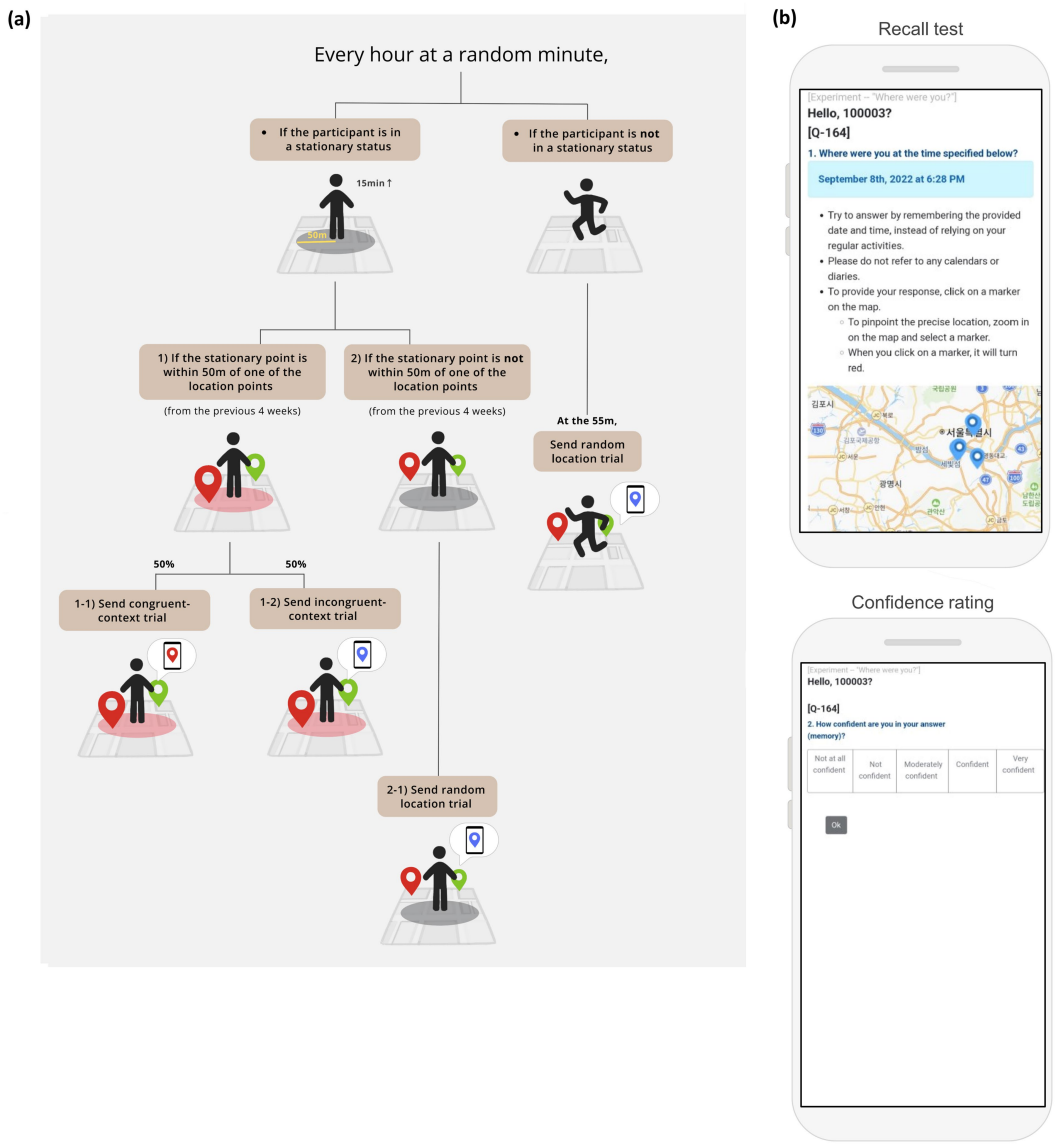
The experimental design of the experiment. **(A)** A diagram of how the test trials were generated, **(B)** display of the recall test, and the confidence rating task on the participant’s smartphone.

To balance the number of trials that match the retrieval context and the encoding context (i.e., congruent-context trial) and that do not match (i.e., incongruent-context trial), each memory test was generated based on the participant’s current location using the following steps (see Figure 1A). First, we examined whether the participant was in a stationary status (i.e., within a 50-meter radius for more than 15 min). Second, if they were in a stationary status, we examined their current location by checking if the stationary point was within 50 meters of one of the location points from the previous 4 weeks. Third, if their current stationary point was one of the locations from the previous 4 weeks, there was a 50% chance of sending a memory test about the current location (i.e., congruent-context trial), and a 50% chance of sending a memory test that was not about the current location, which was randomly chosen (i.e., incongruent-context trial). Fourth, if the stationary point was not one of the locations that were detected during the first 4 weeks of the data collection phase, a memory test about a random location was sent. Finally, if a stationary point was not detected within an hour bin, a memory test about a random location was sent at the 55-min mark.

In each test trial, participants were given a specific time and date. Then, they were asked to indicate their location at a specific date and time by choosing a location marker on a map (i.e., “Where were you at the time specified below?”; see Figure 1B). The test page allowed participants to zoom in and out of the map, with location markers representing location points visited during the data collection phase. Thereafter, participants were asked to rate their confidence on a scale ranging from 1 (not confident at all) to 5 (very confident). Participants were only allowed to submit their responses within 5 min of receiving the test link. Additionally, they were instructed not to use any calendars or diaries but to rely only on the provided date and time information for their recall.

## 3 Results

We excluded three participants who had an accuracy lower than 5% in the memory test, which resulted in 47 participants for the final analyses. We additionally excluded 198 trials (4.63% of the total trials) by filtering out trials that had extreme reaction times (RT), which were outside of the mean ± 2.5SD range. The final data contained 4,072 responses, with an average of 117.32 (*SD* = 18.37) responses per participant. The average proportion of correct responses across participants was 0.48 (*SD* = 16.45) with a mean RT of 17,801 ms (*SD* = 5,160 ms). The average confidence rating was 3.47 (*SD* = 0.50) with a mean RT of 1,690 ms (*SD* = 268 ms).

### 3.1 Context-dependent memory effect

**Figure 2:**
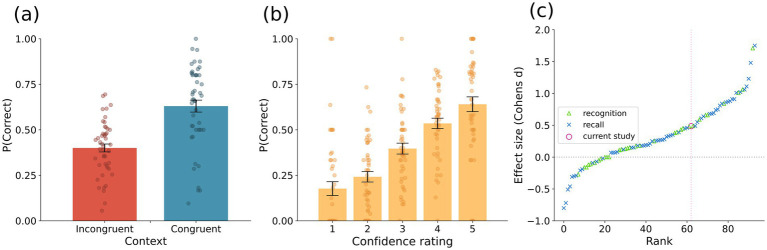
Behavioral results showing the context-dependent memory effect. **(A)** Mean accuracy by context congruent and incongruent conditions. **(B)** Mean accuracy as a function of participants’ confidence ratings. **(C)** Effect size of the context-dependent memory effect compared with previous research reviewed by Smith and Vela (2001). The red O represents the effect size of the current study, the blue X mark and green triangle represent previous studies using a recall and recognition task, respectively. *Error bars represent ± 1 SEM.*

To examine the context-dependent memory effect, we compared the accuracy between the two context conditions (i.e., congruent-context condition and incongruent-context condition) by conducting a logistic mixed-effects analysis using the *lme4* (Bates et al., 2015) package with R (R Core Team, 2023). The congruent-context condition, where the encoding and retrieval contexts matched, was coded as 1, and the incongruent-context condition, where the context at encoding and retrieval was different, was coded as 0 (i.e., context congruency). Correct responses (recalling a location point correctly) were coded as 1, while incorrect responses were coded as 0. We entered the context congruency as a fixed effect, random intercepts for subjects, and by-subject random slopes for the context congruency. A likelihood ratio test indicated that the model including context congruency provided a better fit for the data than a model without it (𝜒^2^ = 48.31, *p* < 0.001). The results for the full model demonstrated a statistically significant association between context congruency and response accuracy rate (*B* = −1.04, *SE* = 0.11, *z* = 9.52, 95% confidence interval = [−1.27, −0.82], *p* < 0.001; see Figure 2A), suggesting that accuracy was higher when the retrieval context matched the encoding context. To examine the relationship between confidence rating and response accuracy rate, we entered confidence rating into the model as a fixed effect, and subject as a random intercept. A likelihood ratio test demonstrated that the model including confidence rating provided a better fit for the data than a model without it (𝜒^2^ = 488.59; *p* < 0.001), revealing that higher confidence ratings were predicted of better memory performance (*B* = 0.70, *SE* = 0.03, *z* = 20.50, 95% confidence interval = [0.64, 0.77], *p* < 0.001; see Figure 2B). We have also examined the effect of context congruency on confidence ratings by entering context congruency as a fixed effect and subject as a random intercept in a linear mixed-effects model. Results showed a statistically significant effect for context congruency (*B* = −0.18, *SE* = 0.03, *t* = −4.89, 95% confidence interval = [−0.26, −0.11], *p* < 0.001) with the model showing a better fit than a model without context congruency (𝜒^2^ = 23.82; *p* < 0.001).

The effect size (Cohen’s *d*) for the context-dependent memory effect was 0.49 (95% confidence interval = [0.42, 0.55]; see Figure 2C). We further compared the magnitude of the effect size with previous studies (Smith and Vela, 2001), which examined the context-dependent memory effect. The magnitude of the effect size in the current study was ranked at the top 31%.

### 3.2 Frequency effect

**Figure 3:**
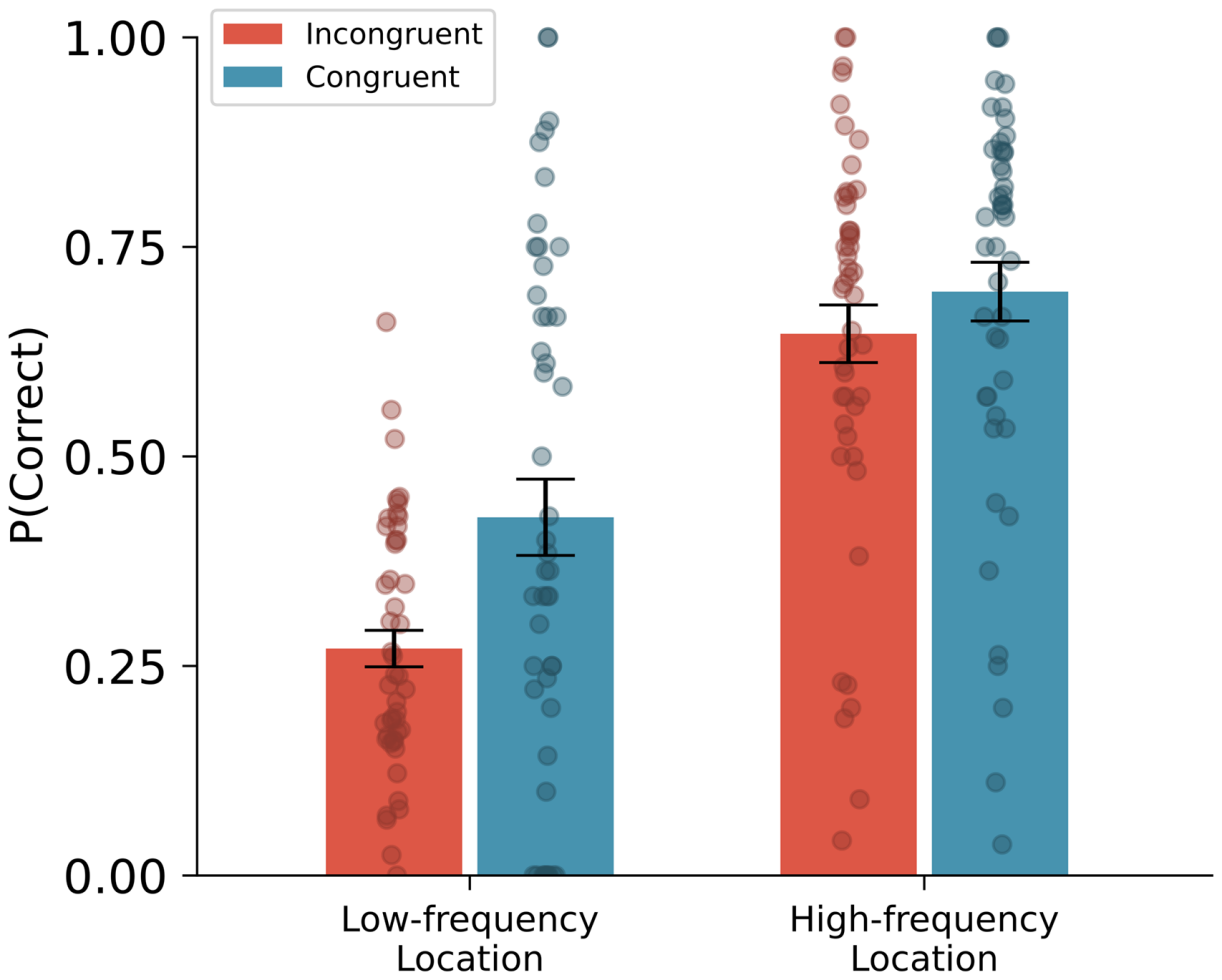
Context-dependent memory effect by location frequency. *Error bars represent ± 1 SEM.*

As the context-dependent memory effect can be mediated by the frequency of the visited locations, we further examined the effect of location-frequency (i.e., how often the participant visited the cued location during the 4-week data collection period) on the context-dependent memory effect. The analysis employed a logistic mixed-effects model. As fixed effects, we entered context congruency, location frequency, and the interactions into the model. As a random effect, we had intercepts for subjects. A likelihood ratio test indicated that the model including interaction terms provided a better fit for the data than a model without it (𝜒^2^ = 13.38, *p* < 0.001). Results again showed a statistically significant effect for context congruency (i.e., context-dependent memory effect; *B* = −0.74, *SE* = 0.08, *z* = 9.10, 95% confidence interval = [−0.90, −0.58], *p* < 0.001), and an effect for location frequency (*B* = 0.72, *SE* = 0.09, *z* = 7.77, 95% confidence interval = [0.55, 0.91], *p* < 0.001), and interaction (*B* = 0.40, *SE* = 0.11, *z* = 3.74, 95% confidence interval = [0.19, 0.61], *p* < 0.001; see Figure 3). We have also examined the effect of location frequency on confidence ratings by entering location frequency as a fixed effect and subject as a random intercept in a linear mixed-effects model. Results showed a statistically significant effect for location frequency (*B* = 0.140, *SE* = 0.02, *t* = 6.14, 95% confidence interval = [0.09, 0.18], *p* < 0.001) with the model showing a better fit than a model without location frequency (𝜒^2^ = 37.59; *p* < 0.001).

To further examine the context-dependent memory effect by location frequency, we median split each participant’s data based on location frequency (i.e., High-frequency location vs. Low-frequency location). Then, we conducted a logistic mixed-effects model with context congruency as fixed effect and subjects as a random effect for each location-frequency data set. Results showed a statistically significant effect of context-dependent memory for both Low-frequency location (*B* = −0.95, *SE* = 0.13, *z* = 7.27, 95% confidence interval = [−1.20, −0.69], *p* < 0.001), and High-frequency location (*B* = −0.26, *SE* = 0.11, *z* = 2.31, 95% confidence interval = [−0.48, −0.04], *p* = 0.02). However, when comparing the effect size, the effect was stronger for the Low-frequency location (Cohen’s *d* = 0.44, 95% confidence interval = [0.32, 0.55]) than for the High-frequency location (Cohen’s *d* = 0.15, 95% confidence interval = [0.06, 0.24]).

### 3.3 Context dwell time effect

We additionally investigated how the context-dependent memory effect changes across time in a given context. For instance, it may take time for the participants to encode the given context, and the context-dependent memory effect may be weak if the participant has only recently entered the given context (e.g., just arrived at a specific environment or location). Therefore, we analyzed the relationship between the duration of time that participants spent in a given context before responding to the memory test (i.e., context dwell time) and the context-dependent memory effect. Here, context was defined as a stationary-point as described in the Methods section (i.e., when the participant stayed within a 50-meter radius for more than 15 min). Using a logistic mixed-effects model, we entered context dwell time and context congruency with interaction terms into the model as fixed effects. As a random effect, we had intercepts for subjects. A likelihood ratio test demonstrated that the model including interaction terms provided a better fit for the data than a model without it (𝜒^2^ = 10.62, *p* = 0.001). The results showed a statistically significant effect for context dwell time (*B* = 0.23, *SE* = 0.07, *z* = 3.25, 95% confidence interval = [0.10, 0.37], *p* = 0.001), context congruency (*B* = −1.02, *SE* = 0.08, *z* = 13.54, 95% confidence interval = [−1.17, −0.87], *p* < 0.001), and their interaction (*B* = −0.25, *SE* = 0.08, *z* = 3.14, 95% confidence interval = [−0.42, −0.10], *p* = 0.002). As there was an interaction effect, for each subject we median split the data by congruent-context and incongruent-context, and conducted separate logistic mixed-effects models with context dwell time as fixed effect and random intercepts for subjects. Results only showed a statistically significant effect for context dwell time in the congruent-context condition (*B* = 0.26, *SE* = 0.08, z = 3.32, 95% confidence interval = [0.11, 0.42], *p* < 0.001), while not in the incongruent-context condition (*B* = −0.03, *SE* = 0.05, z = 0.54, 95% confidence interval = [−0.12, 0.07], *p* < 0.59). Results imply that as the length of time from entering a certain context increases, memory accuracy increases, but only when the context is congruent (i.e., when there is a match between the current context and the context that is trying to be retrieved).

We further expanded the analysis by examining whether context dwell time would have interactions with location frequency. Using a logistic mixed-effects model, we included context dwell time, context congruency, and location frequency with interaction terms into the model as fixed effects. We set intercepts for subjects as random effects. A likelihood ratio test showed that the model with interaction terms better matched the data compared to a model without it (𝜒^2^ = 36.71; *p* < 0.001). Following previous results, there were statistically significant effects for context dwell time (*B* = 0.19, *SE* = 0.07, *z* = 2.71, 95% confidence interval = [0.06, 0.33], *p* = 0.007), context congruency (*B* = −0.62, *SE* = 0.09, *z* = 7.14, 95% confidence interval = [−0.79, −0.45], *p* < 0.001), and location frequency (*B* = 0.70, *SE* = 0.10, *z* = 7.29, 95% confidence interval = [0.52, 0.90], *p* < 0.001). Moreover, there was an interaction between context congruency and location frequency (*B* = 0.62, *SE* = 0.13, *z* = 4.93, 95% confidence interval = [0.38, 0.87], *p* < 0.001) as shown in the previous frequency effect analysis (see Figure 2), whereas the other two-way interactions did not show significant effects (*p* > 0.26).

**Figure 4:**
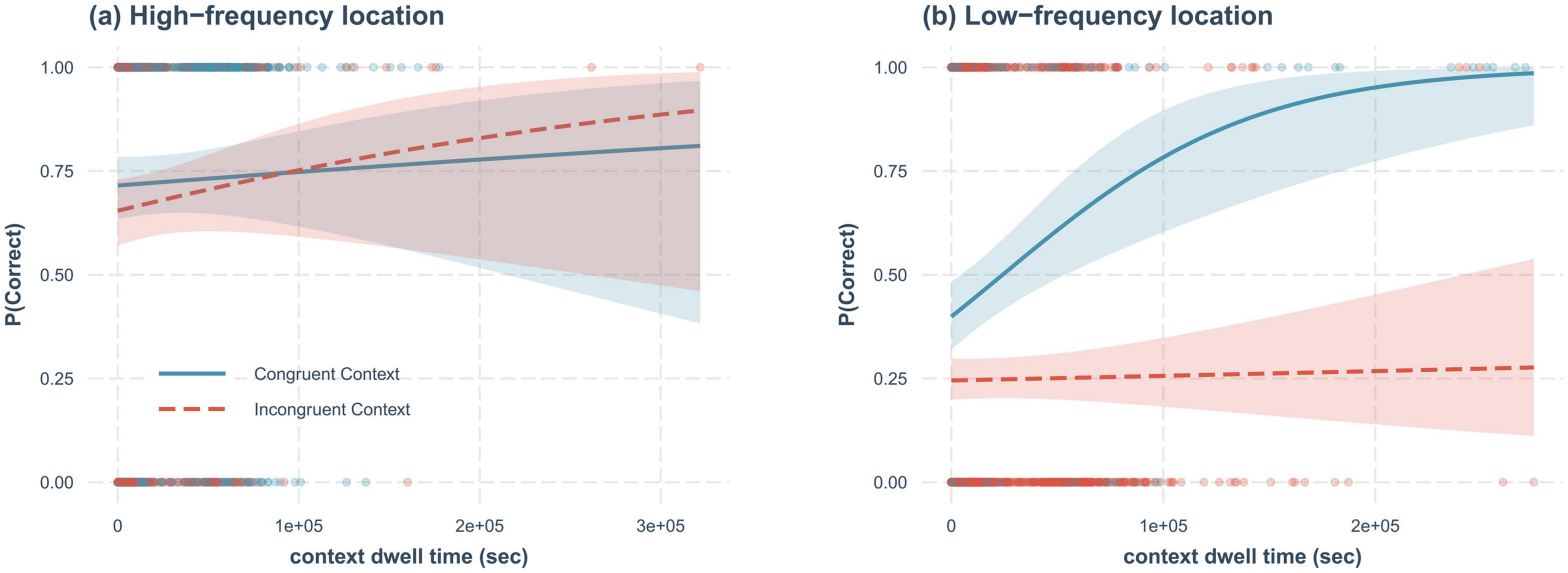
The effects of context dwell time and location frequency on context-dependent memory. **(A)** high-frequency location, and **(B)** low-frequency location. Lines represent the best fitting logistic regression line, and shades represent 95% confidence interval. The red dashed lines and dots represent the trials in the incongruent context, and the blue solid lines and dots represent trials in the congruent context.

Most interestingly, there was a statistically significant effect for a three-way interaction (*B* = 0.62, *SE* = 0.21, *z* = 2.90, 95% confidence interval = [0.20, 1.04], *p* < 0.004). To examine the interactions more closely we median split each participant’s data based on location frequency (i.e., High-frequency location vs. Low-frequency location). Then, for each data set, we conducted logistic mixed-effects models with context dwell time, context congruency, and interaction terms as fixed effects, and intercepts for subjects as random effects. For the High-frequency locations, results only showed a statistically significant effect for context congruency (*B* = −0.23, *SE* = 0.11, *z* = 1.99, 95% confidence interval = [−0.46, −0.001], *p* = 0.047), but not for other effects (*p* > 0.52; see Figure 4A). However, for the Low-frequency locations, there was a statistically significant effect for context dwell time (*B* = 0.52, *SE* = 0.14, *z* = 3.63, 95% confidence interval = [0.27, 0.83], *p* < 0.001), context congruency (*B* = −0.99, *SE* = 0.13, *z* = 7.37, 95% confidence interval = [−1.25, −0.72], *p* < 0.001), and interaction (*B* = −0.50, *SE* = 0.15, *z* = 3.30, 95% confidence interval = [−0.83, −0.23], *p* < 0.001; Figure 4B). The results imply that although there is the context-dependent memory effect regardless of location frequency as shown in the previous analysis, the benefit of the congruent context (i.e., context-dependent memory effect) only increases with context dwell time in the Low-frequency locations.

## 4 Discussion

We investigated the extent to which context influences our daily lives by examining the context-dependent memory effect outside of the laboratory. We used Experience Sampling Methods by utilizing a smartphone app to increase ecological validity, and to further examine the effect in a richer environment. In the experiment, the smartphone app passively collected the GPS location data from the participants over 5 weeks. Thereafter, participants were asked to recall where they were on a particular day and time for two weeks in their daily lives. The results showed evidence for the context-dependent memory effect in the real world with a relatively high effect size (Cohen’s *d* = 0.5) compared to previous studies (Smith and Vela, 2001). The context-dependent memory effect was also shown in the confidence ratings. Moreover, results showed that the context-dependent memory effect was stronger in the low-frequency locations, and became stronger as one dwelled more in a certain context only for the low-frequency location.

Most importantly, we found evidence for the context-dependent memory effect in everyday life, which to our knowledge, has not been previously examined. The experimental design tested the effect in a more natural way, which closely resembled everyday memory retrieval. Compared to previous laboratory-based experiments, the size of the effect was also strong, implying that contextual information has a great impact on our everyday memory retrieval. Although the intention of the current experimental design was to induce a more natural memory encoding environment, the way that the items and context were defined in the study may have generated a more pronounced context-dependent memory effect compared to previous studies. In the current experiment, we have defined the item that one has to remember as a location label (e.g., classroom A), which was later referred to as a location pin on the map, and the context as the attributes of the location (e.g., visual properties of the classroom, ambient sounds and noise in the classroom, etc.). The design was used to generate a more natural encoding phase unlike in a traditional context-dependent memory task, where items (e.g., words) are explicitly presented to the participants, and are independent of the context (e.g., a room with specific properties) that is being manipulated. The interdependent nature of the item and context in the study has been discussed to improve the context-dependent memory effect in previous studies. For example, in a study exploring context-dependent memory through a series of experiments, Fernandez and Glenberg (1985) discuss that one crucial aspect in demonstrating the context-dependent memory effect lies in the association between the item and the context. The stronger the association between the item and context, the more benefit there will be for later retrieval when the context is also presented as a cue. Converging evidence comes from studies that use VR methods (Shin et al., 2021), where they find a stronger context-dependent memory effect when the items are related to the context. Another possible reason for a stronger effect may stem from the fact that the to-be-retrieved items were self-locations. As most of these locations are familiar contexts to the participant compared to the contexts used in the laboratory studies, it is possible that they are more distinguishable from each other, which would generate a stronger effect. The properties of the current experimental design more closely resemble the context-dependent memory effect observed in real life, where attributes are more interconnected compared to those in laboratory studies (e.g., Yim et al., 2024). The frequency effect is another interesting finding. The context-dependent memory effect was shown in both high- and low-frequency locations. However, the effect was stronger in the low-frequency locations. Noting that the overall memory accuracy was higher in the high-frequency locations, it is possible that the accuracy in the high-frequency locations was already at the ceiling and there was no room for the contextual information to contribute. Another possible explanation can be from the fact that low-frequency locations have fewer associated contexts, and individuals may find it easier and less confusing to reinstate the contextual information as there are fewer competing contexts. Relatedly, it is possible that the activities held in these locations are the sources of the frequency effect. For example, low-frequency locations may have more unique activities involved (e.g., mountain – hiking), whereas high-frequency locations may have diverse activities involved (e.g., home – resting, working, eating, etc.). This could either be due to the number of visits influencing the diversity of the activities (i.e., fewer visits involve fewer activities), or it could be that the nature of the location attracts fewer visits (i.e., for activities that occur less frequently such as hiking, there would be fewer visits to such locations). Further examination of the nature of the location and the activities involved would provide a better understanding of the frequency effect. As frequency is a well-known factor that affects memory performance (e.g., Popov and Reder, 2020), the result opens opportunities for further research to investigate other factors that may interact with the effect. Moreover, considering the mixed results from the previous laboratory studies that examined the context-dependent memory effect (Smith and Vela, 2001), it is plausible that insignificant findings are due to confounding factors related to the properties of the stimulus, such as frequency.

The effect of the context dwell time provides a novel contribution to understanding the temporal dynamics of the context-dependent memory effect. The effect of context dwell time was more prominent in the low-frequency location, where the contribution of the matched-context enhanced memory accuracy as the dwell time increased. It is possible that the effect may arise from the time required for contextual information to be encoded and reinstated, which is necessary for effective use during testing and benefiting memory retrieval. Moreover, assuming that contextual information drifts over time (e.g., Howard and Kahana, 2002), contextual representation of the current context would not be representative at the start of a given context as information from the previous context would be lingering and mixed with the incoming contextual information. The results also provide an opportunity to re-think previous laboratory studies where the context (e.g., background color of the screen) is presented with the item (e.g., word) simultaneously at test. It is possible that for the studies that did not find a context-dependent memory effect, a longer dwell time was required for the contextual information to be processed and fully used. Moreover, the results also imply the possibility that the required time for the context information to be processed and used may differ depending on the type of stimulus.

The results of the current study come with a caveat, where we were not able to control the retrieval process that the participants used. Although results showed strong evidence for a context-dependent memory effect and their detailed characteristics, it is possible that the participants did not only rely on direct memory retrieval as the traditional context-dependent memory effect assumes. For example, Friedman (1993) has discussed that there can be several processes that an individual can use when retrieving an event that happened in everyday life. For example, participants can use a distance-based process where they rely on the strength of the event (i.e., recent memories will be stronger than non-recent memories). A location-based process can also be used, where they rely on how well the current cue matches the stored memory. The mechanism that explains the traditional context-dependent memory effect (Tulving and Thomson, 1973) would be a location-based process, where the external context cue aids in retrieving the stored memory. Finally, participants can also use the order information (i.e., relative-time-based process), and infer when an event happened. For example, by knowing that event [B] happens after event [A], and knowing (or remembering) that event [B] happened at time [T], one can infer that event [A] may have happened before time [T]. Similarly, in the current study, it is possible that participants inferred where they were at a certain time based on the knowledge they have obtained through their daily routine (e.g., I am usually at the library in the morning, or I am usually at home after 9 pm). In the current study, it is not possible to parse out the different processes that the participants used as the study design was not intended for that purpose. Instead, it was aimed to capture memory retrieval in the real world setting. However, we acknowledge that participants may have employed different processes highlighting the need for future studies to investigate the impact of these different processes.

Finally, the methodology used in the current study provides a novel way of testing the context-dependent memory effect in a more ecologically valid manner, and further contributes to memory research in real-life settings by utilizing experience sampling methods. Although we acknowledge that a single experiment outside of the laboratory does not perfectly represent what happens in everyday life (Banaji and Crowder, 1989; Morton, 1991), the method allows us to gain a more holistic understanding of individuals’ experiences and examine them in more detail compared to laboratory studies. Assessing individualized frequency in memory enhances the ecological validity of measuring individuals’ genuine memory performance. In addition to increasing ecological validity, the current method also allows us to capture various properties of the effect, such as frequency and context dwell time in memory retrieval, which would have been challenging to obtain in laboratory settings.

## Data Availability

The data presented in the study are deposited in the OSF repository. This data can be found here: https://osf.io/wy5hv/.

## Footnotes

^1^The participants were majorly from the Departments of Data Science, and Cognitive Sciences, where the curriculum does not involve contents regarding human memory nor the context-dependent memory effect. However, we did not ask the participants whether they were aware of the effect.

^2^We did not conduct a proper power analysis, and heuristically estimated that the sample size used Laliberte et al. (2021) would be enough for the current study.

^3^ https://www.traccar.org/
